# MEX3A promotes the malignant progression of ovarian cancer by regulating intron retention in TIMELESS

**DOI:** 10.1038/s41419-022-05000-7

**Published:** 2022-06-17

**Authors:** Fangfang Li, Chen Zhao, Yuchao Diao, Zixiang Wang, Jiali Peng, Ning Yang, Chunping Qiu, Beihua Kong, Yingwei Li

**Affiliations:** 1 Department of Obstetrics and Gynecology, Qilu Hospital of Shandong University, Jinan, Shandong 250012 China; 2 Department of Obstetrics and Gynaecology, Binzhou Medical University Hospital, Binzhou, 256600 Shangdong China; 3 Department of Obstetrics and Gynaecology, The Affiliated Hospital of Qingdao University, Qingdao, 266000 Shangdong China; 4 Medical Integration and Practice Center, Cheeloo College of Medicine, Shandong University, Jinan, Shandong 250012 China

**Keywords:** Ovarian cancer, Ovarian cancer

## Abstract

The latest research shows that RNA-binding proteins (RBPs) could serve as novel potential targets for cancer therapy. We used bioinformatics analysis to screen and identify the key RBPs in ovarian cancer, from which we found that Mex-3 RNA Binding Family Member A (MEX3A) was intimately associated with the clinical prognosis of ovarian cancer. Nevertheless, little is known about its biological roles in ovarian cancer. In this case, we observed that MEX3A was highly overexpressed in fresh-frozen ovarian cancer tissues. MEX3A knockdown suppressed the development and invasion of ovarian cancer cells, while MEX3A overexpression promoted the proliferation and invasion of ovarian cancer cells. Mechanistically, TIMELESS was the critical downstream target gene of MEX3A, as demonstrated through alternative splicing event analysis based on RNA-seq. MEX3A knockdown resulted in retention of intron twenty-three of TIMELESS mRNA and decreased TIMELESS mRNA owing to stimulation of nonsense-mediated RNA decay (NMD). Additionally, we found that TIMELESS overexpression with MEX3A knockdown partially restored the proliferation ability of ovarian cancer cells. The results of this paper demonstrated that the MEX3A/TIMELESS signaling pathway was a key regulator of ovarian cancer, and MEX3A was a novel possible treatment target for ovarian cancer patients.

## Introduction

Ovarian cancer is the second most common cause of death from gynecological malignant tumors among women worldwide [1]. In the global cancer data of 2021, in the US, there are 21,410 new cases of ovarian cancer and 13,770 new deaths [2]. Even in developed countries that have the most advanced medical facilities, patients with ovarian cancer only have a 30–40% 5-year survival rate [3]. More than 70% of ovarian cancer cases are diagnosed at an advanced stage [4]. The high rate of mortality and relatively low rate of survival with ovarian cancer are mainly due to the lack of effective screening methods and the early characteristic clinical symptoms [4]. Although the development of ovarian cancer has advanced in terms of surgery and chemotherapy in recent years, the benefits of these treatments are limited [5]. Therefore, exploring specific markers for the early diagnosis and targeted therapy of ovarian cancer is of great significance to improve the prognosis of ovarian cancer patients.

RNA-binding protein (RBP) is a significant part of posttranscriptional regulation and plays a key role in regulating all kinds of RNA processes, including splicing, transport, degradation of non-coding RNA, and translation [6]. RBP-mediated gene regulation participates in various biological processes of tumor initiation and progression, such as proliferation, metastasis, invasion, and drug resistance [7]. Studies have found that many RBPs are related to the initiation and progression of tumors [8, 9]. For example, SNRPB facilitates the occurrence of non-small cell lung tumors in part by regulating RAB26 [10]. The splicing factor USP39 increases the malignancy of ovarian cancer cells by maintaining efficient splicing of oncogenic HMGA2 [11]. Recent studies have shown that RBPs are an emerging drug target in tumor therapy [12].

MEX3A belongs to the Mex3 family [13] and is a type of RNA-binding protein. MEX3A contains an RNA-binding domain and a C-terminal RING finger domain involved in posttranscriptional regulation [14]. There is evidence that MEX3A is related to the occurrence of cancer [15–18]. MEX3A was overexpressed, and MEX3A knockdown suppressed the proliferation of pancreatic ductal adenocarcinoma cells [15]. MEX3A could interact with LAMA2 to promote lung adenocarcinoma metastasis [16]. MEX3A knockdown restrained the malignant biological advances of esophageal squamous cell carcinoma cells by targeting CDK6 [17]. MEX3A knockdown decreased invasion and proliferation through the RAP1/MAPK pathway in colorectal cancer [18]. Recently, MEX3A was reported to be a prognostic biomarker and served as a novel cancer-critical splicing factor in endometrial cancer [19]. However, the relationship between MEX3A and ovarian cancer is not yet known.

In our research, we analyzed the expression and biological functions of MEX3A in ovarian cancer. The results showed that MEX3A was overexpressed in ovarian cancer tissues and involved in the poor prognosis of ovarian cancer patients. MEX3A promoted the malignant biological progression of ovarian cancer both in vitro and in vivo. In addition, we revealed that MEX3A maintained efficient splicing of oncogenic TIMELESS. Thus, MEX3A has the potential to be a new diagnostic biomarker and therapeutic target for ovarian cancer patients.

## Results

### The overexpression of MEX3A is associated with poor survival in patients with ovarian cancer

**Fig. 1:**
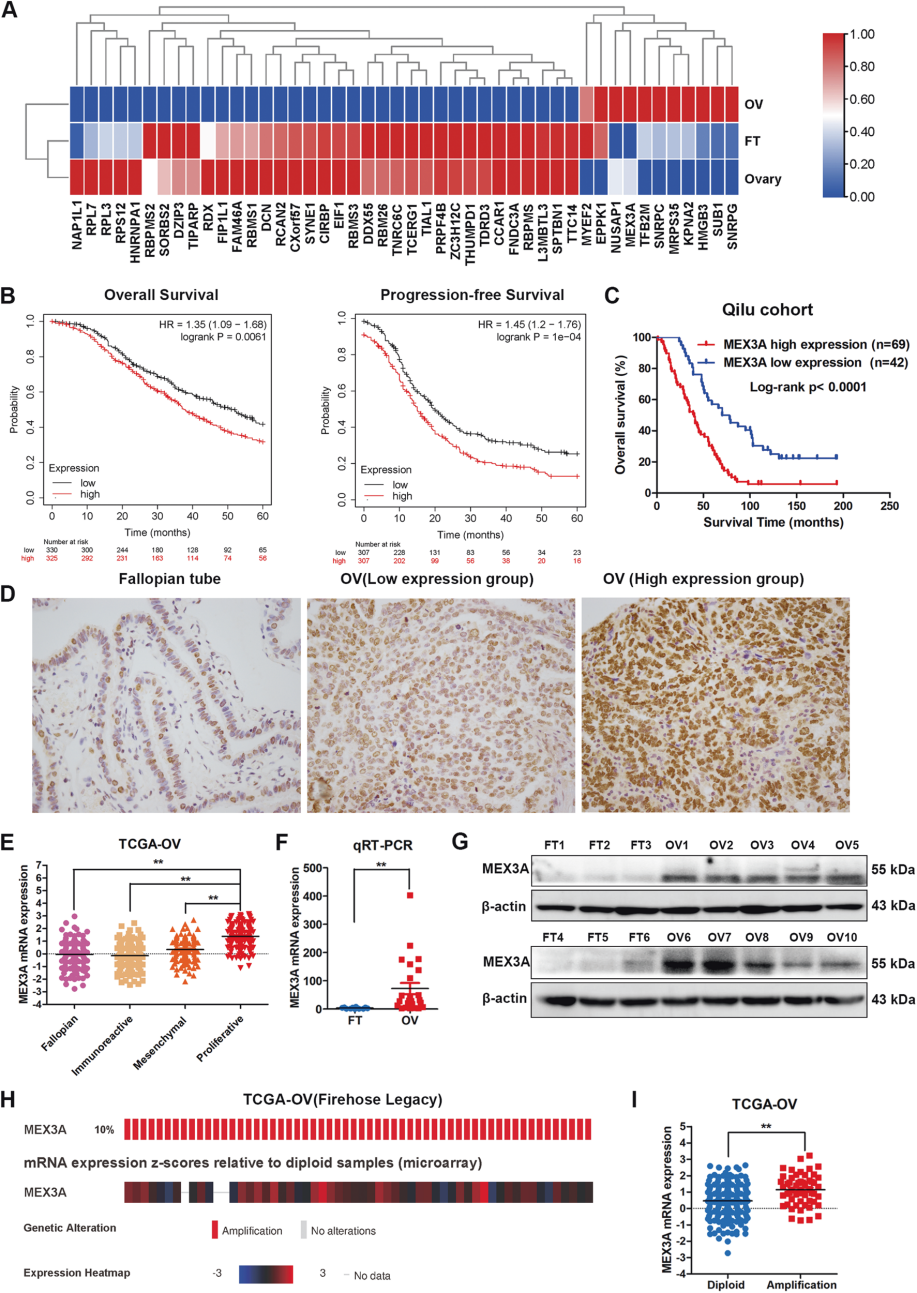
MEX3A is upregulated in ovarian cancer and correlates with poor prognosis. **A** Heatmap showing the expression of 46 differential key RNA binding proteins in ovarian cancer (*n* = 426), normal fallopian tube (FT, *n* = 5) and ovary tissues (*n* = 88) from TCGA-GTEX database. **B** Kaplan–Meier analysis showed the effect of MEX3A expression on the overall survival (High expression group, *n* = 325; Low expression group, *n* = 330) and progression-free survival (High expression group, *n* = 307; Low expression group, *n* = 307) of ovarian cancer patients from Kaplan–Meier Plotter (http://kmplot.com/). **C** Kaplan–Meier analysis showed the effect of MEX3A expression on the overall survival based the Tissue Microarray of high-grade serous ovarian cancer from our tissue bank. **D** Immunohistochemical staining images of MEX3A in fallopian tube and ovarian cancer based the Tissue Microarray of high-grade serous ovarian cancer from our tissue bank. **E** The relative expression of MEX3A in ovarian cancer, according to the current molecular typing of ovarian cancer from TCGA-OV. (Fallopian, *n* = 134; Immunoreactive, *n* = 108; Mesenchymal, *n* = 106; Proliferative, *n* = 135). **F** qRT-PCR analysis of MEX3A mRNA expression between ovarian cancer (*n* = 24) and fallopian tube (*n* = 12) tissues (*n* = 4 biologically independent samples). **G** Western blotting showed the differences in the MEX3A protein levels between ovarian cancer (*n* = 10) and fallopian tube (*n* = 6) tissues. **H** Genetic alterations of MEX3A in ovarian cancer from TCGA cohort (Firehose Legacy, *n* = 579). **I** Relative MEX3A mRNA expression of samples with different copy number variation status from TCGA. (Non-amplified group, *n* = 190; amplified group, *n* = 55). *P*-value was obtained by Log-rank test (**B**, **C**) or unpaired *t*-test (**E**, **F**, and **I**). *P < 0.05, **P < 0.01.

To search for the key RNA-binding proteins in ovarian cancer, we sifted the differentially expressed genes between ovarian cancer and normal ovary tissues from TCGA cohort through GEPIA ( | Log2FC | ≥1, *q* < 0.01) (http://gepia.cancer-pku.cn/), and we found that 7638 genes were differentially expressed, including 2622 upregulated genes and 5016 downregulated genes. Zhao et al. reported a high-priority list of 145 RBP genes associated with ovarian cancer aggressiveness [20]. Overlapping analysis between 7638 differentially expressed genes and these 145 RBPs identified 46 key RBPs that were involved in the initiation and development of ovarian cancer. The expression of 46 RBPs was further validated through TCGA-GTEX data. The study showed that 35 genes were under-expressed, and nine genes were overexpressed in ovarian cancer compared with normal fallopian tube and ovary tissues (Fig. 1A). The effect of 9 upregulated RBPs on clinical prognosis was evaluated by Kaplan–Meier Plotter (http://kmplot.com/), and only MEX3A was found to be associated with worse overall survival (OS) and progression-free survival (PFS) of ovarian cancer (Fig. 1B). Immunohistochemistry staining assay was performed to further evaluate MEX3A expression in ovarian cancer and normal fallopian tube tissues based the Tissue Microarray (TMA) of high-grade serous ovarian cancer from our tissue bank. The results showed that MEX3A expression was mainly focused in the nucleus, and MEX3A expression was higher in ovarian cancer than that in normal fallopian tube tissues. High expression group of MEX3A accounted for 62.16% of all the ovarian cancer samples, and high level of MEX3A correlated with ascites volume and poor overall survival (Table S3 and Fig. 1C). Representative images of immunohistochemical (IHC) staining of MEX3A in TMA were shown in Fig. 1D. The differential expression of MEX3A between ovarian cancer and normal tissues is presented in Supplementary Fig. 1A. Moreover, we analyzed the expression of MEX3A in ovarian cancer with different molecular subtypes from TCGA cohort from cBioPortal for cancer genomics(http://www.cbioportal.org/), and the results suggested that MEX3A expression was the highest in the Proliferation subtype, followed by the Mesenchymal type, compared with Fallopian and Immunoreactive subtypes, indicating that MEX3A may be closely related to the proliferation and metastasis of ovarian cancer (Fig. 1E). For the purpose of the expression abundance of MEX3A in fresh-frozen ovarian cancer tissues, we verified MEX3A mRNA expression in 12 cases of normal fallopian tube and 24 cases of ovarian cancer by qRT-PCR and found that MEX3A was highly expressed in ovarian cancer compared with normal fallopian tube tissues (Fig. 1F). We also detected MEX3A protein expression in ten cases of ovarian cancer and 6 cases of normal fallopian tube fresh tissue by western blotting. The results showed that the expression of MEX3A protein in ovarian cancer tissue was significantly higher than that in normal fallopian tube tissues (Fig. 1G).

MEX3A copy number amplification occurred in 10% of ovarian cancer samples in TCGA cohort (Fig. 1H), and MEX3A mRNA expression increased in patients with amplification (Fig. 1I). Through the analysis of TCGA and CCLE databases, the results showed that there was a linear positive relationship between MEX3A amplification and its mRNA expression in both ovarian cancer tissues and cell lines (Supplementary Fig. 1B, C).

### MEX3A knockdown inhibited the proliferation of ovarian cancer cells in vitro and in vivo

**Fig. 2:**
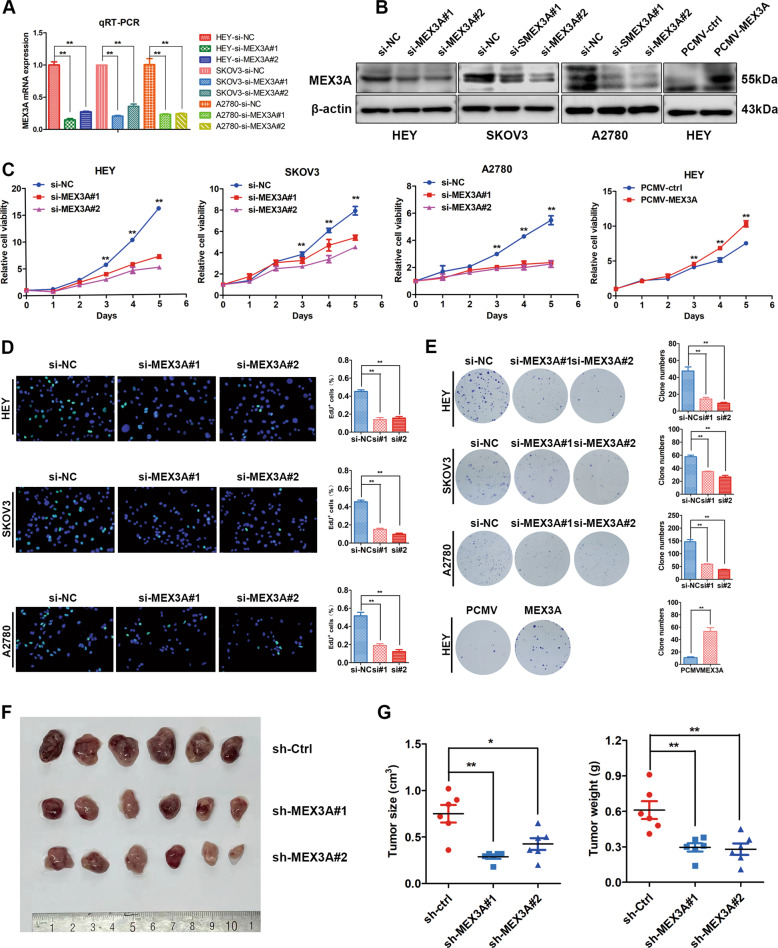
MEX3A knockdown inhibits the cell proliferation and tumor growth of ovarian cancer cells in vitro and in vivo. **A**, **B** MEX3A knockdown and overexpression efficiency were confirmed by qRT-PCR (*n* = 4 biologically independent samples) and western blotting. **C** Growth curve showing the growth of ovarian cancer cells upon MEX3A knockdown or overexpression (*n* = 5 biologically independent samples). **D** EdU assay showed the proliferation ability changes of ovarian cancer cells after MEX3A knockdown (*n* = 5 biologically independent samples). **E** The clonogenic assay showed the clonogenic capacity changes of ovarian cancer cells after MEX3A knockdown and overexpression (*n* = 5 biologically independent samples). **F** Images of tumors isolated from subcutaneous implantation of nude mice with MEX3A knockdown and control cells (*n* = 6 mice per group). **G** Tumor size and weight statistics. *P*-value was obtained by unpaired *t*-test. Results represent the mean ± SD of three independent experiments. *P < 0.05, **P < 0.01.

To verify the function of MEX3A in ovarian cancer cells, we devised two kinds of small interfering RNAs (siRNAs) to downregulate MEX3A expression and constructed MEX3A overexpression cell lines by lentivirus transfection in ovarian cancer cells. MEX3A knockdown and overexpression efficiency were verified by qRT-PCR and western blotting (Fig. 2A, B). Then, we performed growth curve and EdU assays to evaluate the effect of MEX3A on the proliferation ability of ovarian cancer cells, and we found that MEX3A inhibition in HEY, SKOV3, and A2780 cells led to a decrease in the proliferation rate, while overexpression of MEX3A significantly accelerated the growth rate of HEY cells (Fig. 2C, D). The clonogenic assays revealed that MEX3A knockdown resulted in a decrease in clonogenicity capacity, while MEX3A overexpression significantly promoted the clonogenicity capacity of HEY cell lines compared to the empty vector control (Fig. 2E). Moreover, the results of subcutaneous implanted tumors in female nude mice suggested that MEX3A knockdown remarkably inhibited the tumor growth of ovarian cancer cells and led to a decrease in tumor weight and volume (Fig.2F, G). These results implied that MEX3A knockdown reduced the cell proliferation and tumor growth of ovarian cancer cells in vitro and in vivo.

### MEX3A downregulating suppresses the metastasis of ovarian cancer cells

**Fig. 3:**
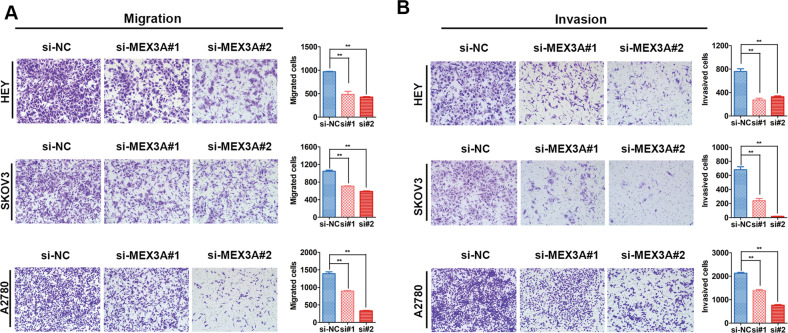
MEX3A downregulating suppresses the metastasis of ovarian cancer cells. **A** Transwell assays were used to evaluate the effect of MEX3A expression on the migration of ovarian cancer cells (*n* = 5 biologically independent samples). **B** Transwell assays were used to evaluate the effect of MEX3A expression on the invasion of ovarian cancer cells (*n* = 5 biologically independent samples). *P*-value was obtained by unpaired *t*-test. Results represent the mean ± SD of three independent experiments. *P < 0.05, **P < 0.01.

Moreover, we used a Transwell assay to determine the roles of MEX3A in the metastasis of ovarian cancer cells. MEX3A knockdown was found to decrease the invasion and migration abilities of ovarian cancer cells (Fig. 3A, B). The study showed that MEX3A promotes the metastasis capacity of ovarian cancer cells.

### Identification of key downstream target genes of MEX3A through RNA-seq

**Fig. 4:**
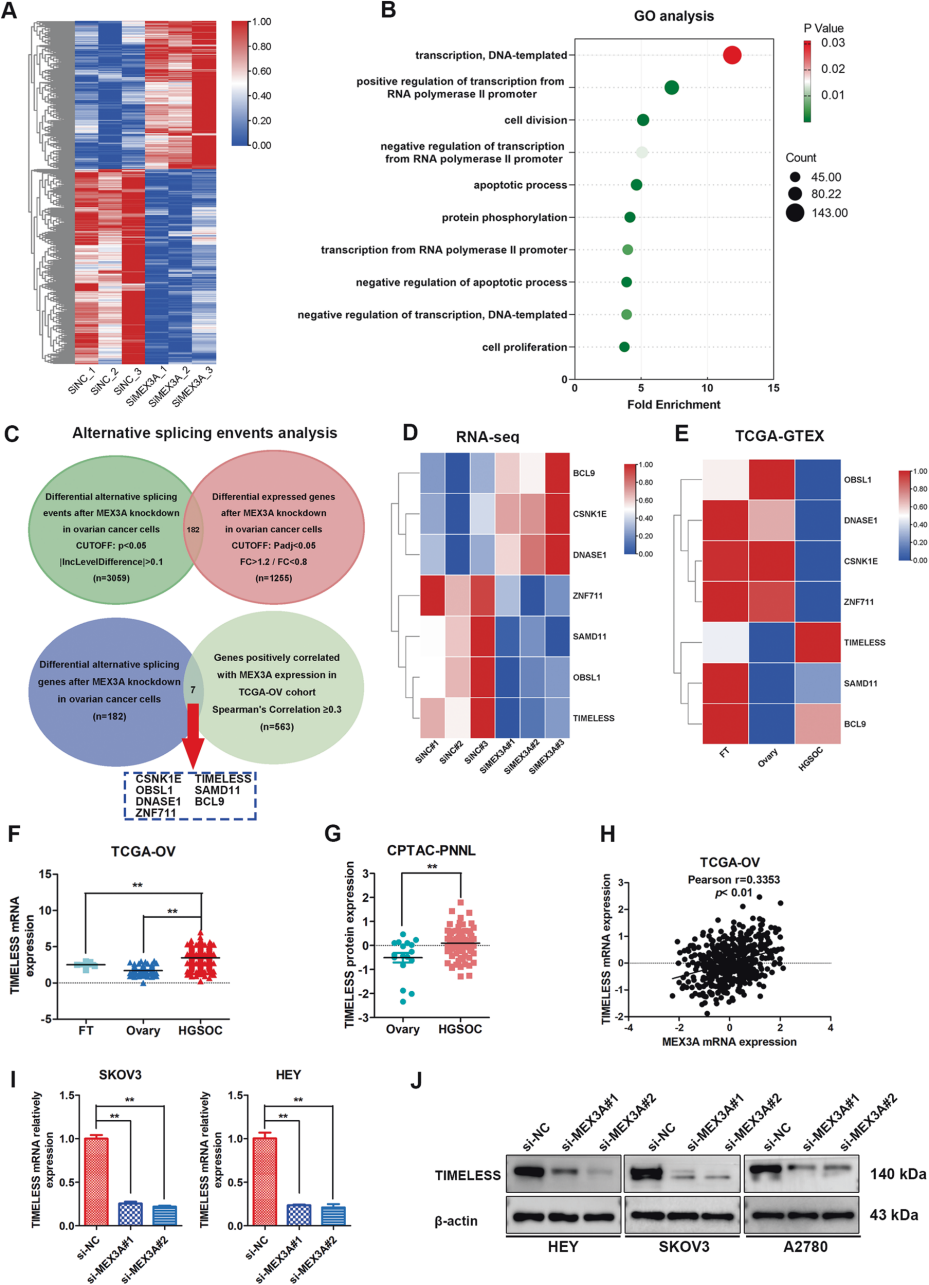
Identification of key downstream target genes of MEX3A through RNA-seq. **A** Heatmap of differentially expressed genes from RNA-seq analysis of MEX3A knockdown in A2780 cells. **B** Bubble diagram showing the results of GO analysis of differentially expressed genes described in (**A**). **C** Venn diagram showing the key DEGs involved in critical alternative splicing events. **D** Heatmap of differential expression of selected genes (*n* = 7) from RNA-seq analysis after MEX3A knockdown. **E** The differential expression of selected genes (*n* = 7) in ovarian cancer (*n* = 426) compared to normal ovaries (*n* = 88) and fallopian tubes (*n* = 5) through TCGA-GTEX database. **F**, **G** Relative mRNA and protein expression of TIMELESS from TCGA ovarian cancer (Fallopian tubes, *n* = 5; Ovary, *n* = 88; HGSOC, *n* = 426) and CPTAC databases (Ovary, *n* = 17; HGSOC, *n* = 77). **H** Correlation analysis between MEX3A and TIMELESS mRNA in ovarian cancer tissues from TCGA ovarian cancer (*n* = 489). **I** qRT-PCR analysis of the relative mRNA expression of TIMELESS in ovarian cancer after MEX3A knockdown (*n* = 4 biologically independent samples). **J** western blotting showed the MEX3A and TIMELESS protein levels after MEX3A downregulation. *P*-value was obtained by unpaired *t*-test (**F**, **G**, and **I**). Results represent the mean ± SD of three independent experiments. *P < 0.05, **P < 0.01.

To study the mechanism by which MEX3A promotes the malignant biological progression of ovarian cancer, we performed RNA-seq after MEX3A knockdown in A2780 cells to analyze the changes in differentially expressed genes (DEGs) between the MEX3A-NC group and the si-MEX3A#1 group (Fig. 4A). We identified 543 upregulated and 712 downregulated genes through differential expression analysis (FC > 1.2/FC < 0.8, Padj < 0.05). Gene enrichment analysis showed that DEGs were mainly related to biological processes such as cell proliferation, apoptosis and transcription regulation (Fig. 4B).

We further analyzed the alternative splicing events that changed after MEX3A knockdown, and the results showed that exon skipping (SE) was the most common event (70%) of all alternative splicing events, followed by intron retention (IR) events (10%). Other alternative splicing types accounted for 20%. To screen the key critical alternative splicing events, we set the threshold (*p* < 0.05, |IncLevelDifference |>0.1) and found 3059 alternative splicing events (1929 SE, 230 IR, 408 MXE, 244 A3SS, and 249 A5SS). To determine the key DEGs involved in critical alternative splicing events, overlapping analysis of DEGs after MEX3A knockdown and 3059 alternative splicing events identified 182 DEGs (Fig. 4C). Furthermore, correlation analysis between 182 DEGs and MEX3A was applied using TCGA ovarian cancer data to obtain 7 DEGs (CSNKE1, OBSL1, DNASE1, ZNF711, TIMELESS, SAMD11, and BCL9) that were positively associated with MEX3A expression (Spearman’s correlation≥0.3). The differential expression of 7 DEGs after MEX3A knockdown is presented in Fig. 4D through RNA-seq analysis. The differential expression of 7 DEGs in ovarian cancer, normal fallopian tube (FT), and ovary tissues was analyzed using the data from TCGA-GTEX database, and the results showed that only TIMELESS was overexpressed, and OBSL1, DNASE1, CSNK1E, and ZNF711 were downregulated in ovarian cancer compared with normal FT and ovary tissues (Fig. 4E). TIMELESS was reported to be aberrantly expressed and intimately associated with the development and progression of human cancers [21–23], and therefore we selected TIMELESS as the key target of MEX3A in ovarian cancer. The differential expression of TIMELESS mRNA between ovarian cancer and normal tissues from the TCGA-GTEX database is shown in Fig. 4F. TIMELESS protein expression was also found to be highly overexpressed in ovarian cancer using the data from CPTAC (Clinical Proteomic Tumor Analysis Consortium) (Fig. 4G). Correlation analysis between MEX3A and TIMELESS verified that TIMELESS expression was positively correlated with MEX3A expression in ovarian cancer tissues (Fig. 4H). Furthermore, qRT-PCR and western blotting were used to evaluate TIMELESS expression after MEX3A knockdown in ovarian cancer cells, and we found that TIMELESS mRNA and protein expression was obviously decreased after MEX3A inhibition (Fig. 4I, J). These results suggested that TIMELESS was the key downstream target involved in the splicing regulation of MEX3A.

### TIMELESS mediates the growth and metastasis of ovarian cancer cells

**Fig. 5:**
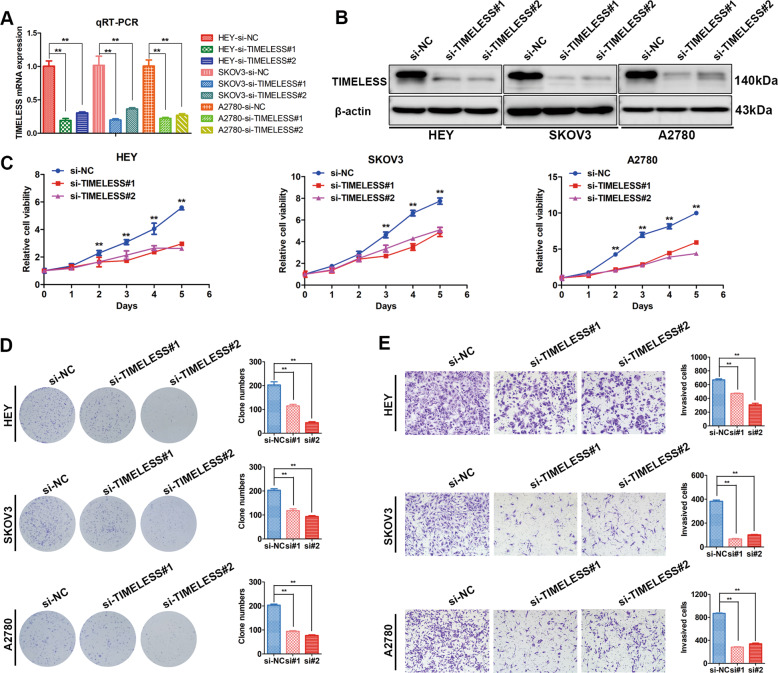
TIMELESS mediates the growth and metastasis of ovarian cancer cells. **A**, **B** TIMELESS knockdown efficiency was confirmed by qRT-PCR (*n* = 4 biologically independent samples) and western blotting. **C** Growth curves and **D** clonogenic assays were used to evaluate the effect of TIMELESS knockdown on the proliferation of ovarian cancer cells (*n* = 5 biologically independent samples). **E** Transwell assays showed the invasion capacity of ovarian cancer cells upon TIMELESS knockdown (*n* = 5 biologically independent samples). *P*-value was obtained by unpaired *t*-test. Results represent the mean ± SD of three independent experiments. *P < 0.05, **P < 0.01.

Next, we verified the biological function of TIMELESS in ovarian cancer cells. Similarly, we designed two small interfering RNAs (siRNAs) to downregulate TIMELESS expression. qRT-PCR and western blotting were used to verify the knockdown efficiency. The results showed that the mRNA and protein expression levels of TIMELESS were decreased by siRNA-mediated downregulation of MEX3A (Fig. 5A, B).

Growth curve analysis showed that TIMELESS knockdown significantly inhibited the proliferation of HEY, SKOV3, and A2780 cells (Fig. 5C). In addition, TIMELESS downregulation also suppressed the clone formation ability of ovarian cancer cells (Fig. 5D). The results of the Transwell experiments showed that invasion capacity decreased after TIMELESS was downregulated (Fig. 5E). These results indicated that TIMELESS could promote the proliferation and metastasis of ovarian cancer.

### TIMELESS knockdown reversed the oncogenic effects caused by MEX3A overexpression

**Fig. 6:**
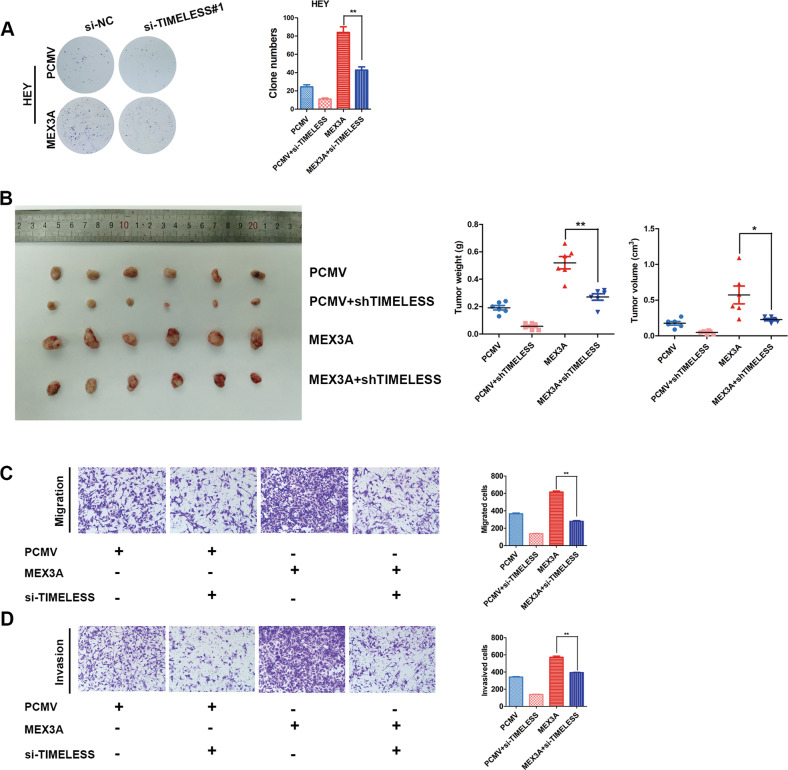
TIMELESS knockdown reversed the biological effects caused by MEX3A overexpression. **A** TIMELESS knockdown reduced the colony formation abilities of MEX3A-overexpressing HEY cells (*n* = 5 biologically independent samples). **B** Xenograft experiments showed TIMELESS knockdown partially reversed the effect of MEX3A overexpression on the growth of ovarian cancer cell in vivo (*n* = 6 mice per group). **C**, **D** TIMELESS inhibition partially reversed the effect of MEX3A overexpression on cell migration and invasion (*n* = 5 biologically independent samples). *P*-value was obtained by unpaired *t*-test. Results represent the mean ± SD of three independent experiments. *P < 0.05, **P < 0.01.

To confirm the function of TIMELESS in MEX3A-mediated proliferation, migration, and invasion of ovarian cancer cells, we transiently transfected TIMELESS siRNA into HEY cells overexpressing MEX3A. TIMELESS knockdown reduced the colony formation abilities of MEX3A-overexpressing HEY cells (Fig. 6A). Consistent with the results of colony formation assays in vivo, xenograft experiments showed TIMELESS knockdown could partially reverse the effect of MEX3A overexpression on the growth of ovarian cancer cells in vivo (Fig. 6B). Moreover, TIMELESS inhibition partially reversed the effect of MEX3A overexpression on the abilities of cell migration and invasion (Fig. 6C, D). Therefore, TIMELESS is the main target that mediates the role of MEX3A in the growth and metastasis of ovarian cancer cells.

### MEX3A regulates the expression of TIMELESS mRNA in an NMD-dependent manner

**Fig. 7:**
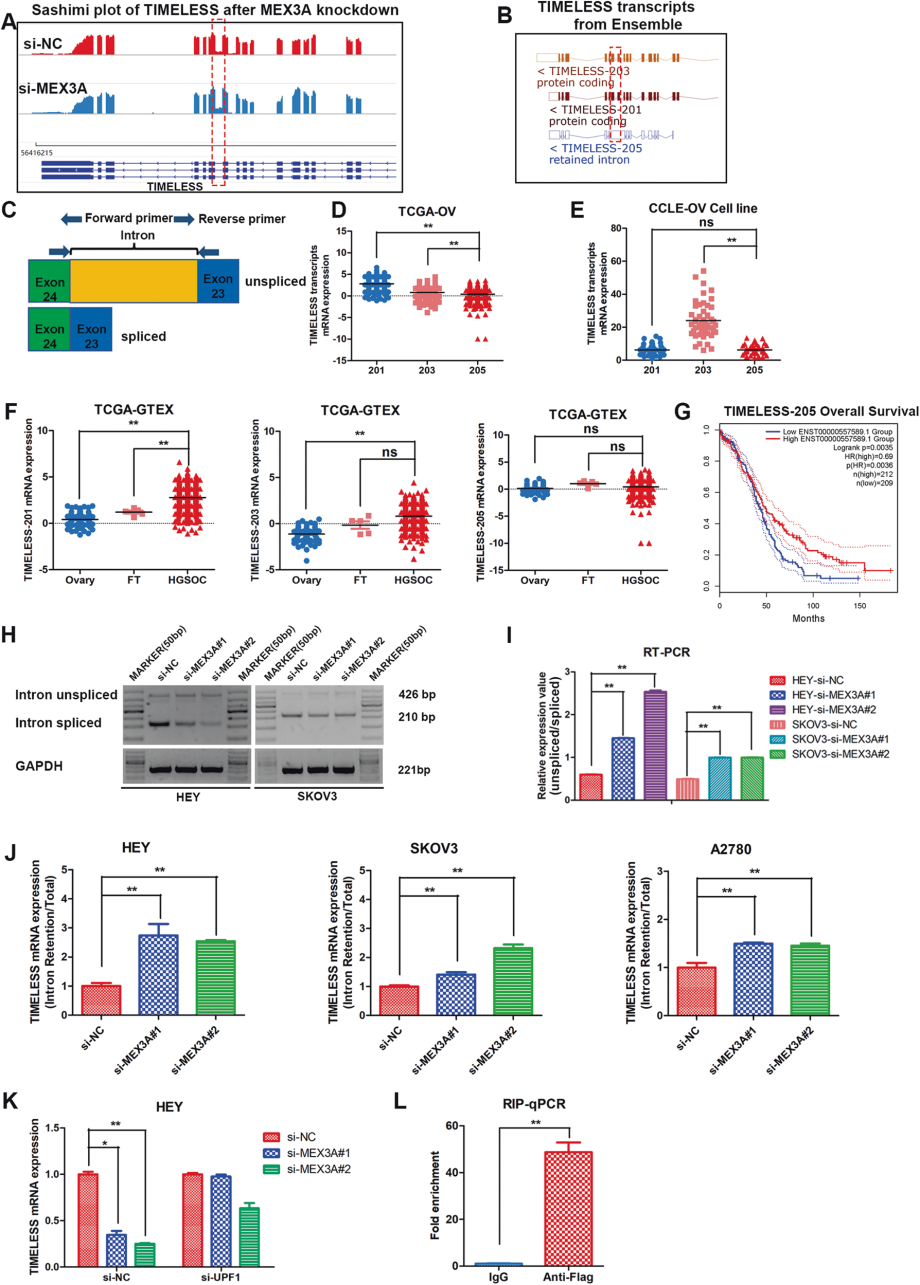
MEX3A regulates the expression of TIMELESS mRNA in an NMD-dependent manner. **A** Sashimi plot visualization of RNA-seq reads mapped to TIMELESS in A2780 cells after MEX3A knockdown. **B** Schematic showing three splicing variants of the TIMELESS mRNA transcript, as identified from the Ensemble genome browser. **C** Schematic diagram showing the position of intron retention and primers used for RT-PCR. **D**–**F** Relative mRNA expression of TIMELESS and its splicing variants described from TCGA, GTEX and CCLE databases. (CCLE, *n* = 47; Fallopian tubes, *n* = 5; Ovary, *n* = 88; HGSOC, *n* = 426) **G** Kaplan–Meier analysis of the effect of TIMELESS-205 expression on the overall survival of ovarian cancer patients from GEPIA (High expression, *n* = 212; Low expression, *n* = 209). **H**, **I** RT-PCR was used to analyze the splicing isoforms of TIMELESS in ovarian cancer cells with MEX3A knockdown (*n* = 3 biologically independent samples). **J** qRT-PCR analysis of the proportion of TIMELESS intron retained transcripts after downregulation of MEX3A in ovarian cancer cell lines using specific intron 23 primers (*n* = 4 biologically independent samples). **K** HEY cells were cotransfected with specific MEX3A siRNA or control and UPF1 siRNA. TIMELESS expression was examined by qRT-PCR (*n* = 4 biologically independent samples). **L** RIP-qPCR was applied to determine the interaction between MEX3A protein and TIMELESS mRNA in MEX3A overexpressed HEY cells using Flag antibody (*n* = 5 biologically independent samples). *P*-value was obtained by unpaired *t*-test. Results represent the mean ± SD of three independent experiments. *P < 0.05, **P < 0.01.

To reveal the mechanism by which MEX3A regulates TIMELESS expression in ovarian cancer, we observed intron 23 retention of TIMELESS mRNA based on Sashimi plot analysis according to the RNA-seq results after MEX3A knockdown (Fig. 7A). Moreover, we analyzed splice variants of the MEX3A mRNA transcript based Ensembl Genome Browser, as shown schematically in Fig. 7B. The results showed that intron 23 was retained between exons 23 and 24 in the noncoding TIMELESS-205 transcript variant (unspliced) compared with the protein-coding transcript variant TIMELESS-201 and 203 (spliced). Retained intron 23 of TIMELESS could produce premature termination of codon in the normal coding region of TIMELESS. Specific primers were designed within exons 23 and 24 that span intron 23 to distinguish whether intron 23 was spliced or not after MEX3A knockdown (Fig. 7C). In addition, we studied the expression of different TIMELESS transcripts in ovarian cancer in the TCGA cohort, and we found that TIMELESS-201 and TIMELESS-203 expression was higher than TIMELESS-205 and that TIMELESS-201 expression was the highest in ovarian cancer tissues (Fig. 7D). However, TIMELESS-203 expression was higher than TIMELESS-205 expression, and there was no difference between TIMELESS-201 and TIMELESS-205 expression in ovarian cancer cell lines from the CCLE database (Fig. 7E). TIMELESS-201 and TIMELSS-203 were overexpressed in ovarian cancer, and there was no difference in TIMELSS-205 expression between ovarian cancer and normal control tissues (Fig. 7F). TIMELESS-205 expression was related to better prognosis in ovarian cancer (Fig. 7G). RT-PCR was applied to determine the relative expression of TIMELESS transcripts using primers that span intron 23 after MEX3A knockdown in ovarian cancer cells, and the results showed that spliced transcript TIMELESS-201/203 expression was decreased and unspliced transcript TIMELESS-205 expression was increased (Fig. 7H). The proportion of unspliced transcript expression compared with spliced transcript expression was obviously increased after MEX3A knockdown (Fig. 7I). Specific primers within intron 23 were also used to validate TIMELESS relative expression after MEX3A knockdown through qRT-PCR, and the results were in accordance with the RT-PCR results (Fig. 7J).

Intron-preserving mRNA transcripts contain early termination codons, which could destroy the open reading frame and trigger nonsense-mediated decay (NMD) degradation. NMD is a quality control monitoring mechanism for the degradation of abnormal mRNA. To further demonstrate whether the NMD pathway was involved in the regulation of TIMESLESS transcripts induced by MEX3A, we downregulated the key factor UPF1 of NMD with siRNA in HEY cells. qRT-PCR was used to evaluate TIMELESS expression after UPF1 knockdown, and the results showed that suppression of UPF1 resulted in a significant increase in TIMELESS mRNA compared with control siRNA-treated MEX3A knockdown in HEY cells (Fig. 7K). More importantly, RIP-qPCR revealed that TIMELESS mRNA expression in the MEX3A overexpression precipitates was much higher comparing with IgG control (Fig. 7L), indicating that the MEX3A protein could bind with TIMELESS mRNA.

**Fig. 8:**
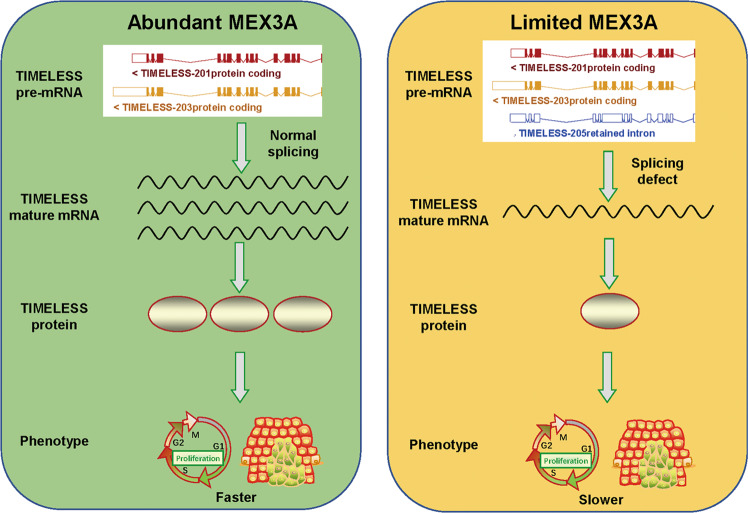
Model showing the effect of MEX3A/TIMELESS signaling on the malignancy of ovarian cancer cells. Abundant MEX3A regulates TIMELESS expression and performs oncogenic functions through maintaining the normal splicing of TIMELESS. At the deficiency of MEX3A, TIMELESS splicing goes wrong which leads to reduction of TIMELESS and decreases the oncogenic ability of MEX3A.

The study showed that the decrease in MEX3A results in the splicing of TIMELESS precursor mRNA into a noncoding transcriptional variant, which is degraded by NMD (Fig. 8).

## Discussion

Aberrant expression and location of RBPs will not only raise the expression of oncogenes but also reduce the expression of tumor suppressor genes, thus contributing to tumorigenesis [12]. In recent years, people have paid increasing attention to the role of RBPs in cancer, and RBPs are anticipated to be a target for cancer treatment. Related studies on RNA-binding proteins have found that the splicing factor SF3B1 accelerates the growth of endometrial cancer cells by controlling KSR2 RNA maturation [24]. SRSF3 regulates the cell growth and maintenance energy metabolism of colon cancer cells through PKM splicing [25]. RBM47 regulates cell fate decisions through the p53/p21 signaling axis [26]. In this study, we revealed that MEX3A was overexpressed in ovarian cancer tissues and correlated with worse prognosis of ovarian cancer patients. MEX3A accelerated the malignant biological behavior of ovarian cancer. TIMELESS was identified as a key downstream target of MEX3A. MEX3A-mediated intron 23 retention in the TIMELESS mRNA to regulate its expression through the NMD pathway.

MEX3A is a member of the MEX3 family. MEX3 is extensively found in mammalian cells. It encodes four different genes, namely, MEX3A, MEX3B, MEX3C, and MEX3D [16]. To date, there are few reports about the effect of MEX3A on tumor cells. Recent research shows that MEX3A is highly expressed in a variety of cancers and promotes the occurrence and metastasis of cancer, which is related to the poor prognosis of patients [27–29]. MEX3A might regulate immune infiltration in the microenvironment of ovarian cancer cells and is a potential therapeutic target [30]. However, MEX3A was reported to suppress proliferation and EMT by restraining the Akt signaling pathway in cervical cancer [31]. These results revealed that MEX3A performs a variety of biological functions in different tumors.

Our analysis of MEX3A expression in clinical samples and open access online database supported that MEX3A was significantly upregulated in ovarian cancer, and high levels of MEX3A in ovarian cancer patients were associated with shorter survival time. A series of experiments confirmed that MEX3A gene knockdown could inhibit the proliferation and invasion of ovarian cancer cells. In contrast, the upregulation of MEX3A promoted the malignant biological behavior of ovarian cancer cells. These results were consistent with the study of MEX3A in the majority of human cancers. Therefore, our research showed that MEX3A served as an oncogene in ovarian cancer.

Dysregulation of alternative splicing is judged to be a hallmark of tumors. MEX3A was reported to correlate with RNA splicing in endometrial cancer by analyzing RNA-seq data [19]. The role of MEX3A in alternative splicing regulation was unclear in ovarian cancer until now. In this study, we analyzed RNA-seq data after MEX3A knockdown in ovarian cancer cells and identified the critical downstream target TIMELESS involved in the alternative splicing regulation of MEX3A. Our study showed that MEX3A inhibition suppressed the alternative splicing of intron 23 between exons 23 and 24 and decreased TIMELESS expression by activating the NMD pathway.

TIMELESS can regulate daily rhythms, but the most important features of TIMELESS are DNA replication and damage repair [32]. Recent research has shown that TIMELESS plays an important role in cancer progression. TIMELESS promoted breast cancer progression by activating MYC [33] and regulated cancer cell progression via the Sp1/ACER2/S1P axis [23]. TIMELESS depletion in cervical cancer can inhibit its proliferation and improve cisplatin sensitivity [34]. TIMELESS was also reported to serve as a tumor suppressor and inhibit breast cancer cell invasion and metastasis by knocking down the expression of MMP9 [35].

This study revealed that TIMELESS knockdown inhibited the proliferation, invasion and metastasis of ovarian cancer cells. TIMELESS knockdown impaired phenotypic changes in ovarian cancer cells caused by MEX3A overexpression. We identified TIMELESS as an important target involved in MEX3A in mediating the growth and metastasis of ovarian cancer cells. Moreover, we revealed that TIMELESS is a new target for MEX3A-mediated RNA maturation. Above all, we identified that TIMELESS contributed to MEX3A’s carcinogenic function in ovarian cancer. However, the detailed regulatory mechanisms of MEX3A regulating TIMELESS was not fully revealed, and it was still unknown whether the regulation was dependent on HNRNPK domain or not [36]?

This was the first study to evaluate the expression of MEX3A and its relationship with clinical prognosis and parameters using Tissue Microarray of high-grade serous ovarian cancer. The results showed that MEX3A expression was significantly increased in ovarian cancer tissues compared to normal fallopian tubes. Kaplan–Meier analysis revealed that patients with higher expression of MEX3A (62.16%) correlated positively with poor prognosis for patients with ovarian cancer. Therefore, MEX3A was an independent prognostic biomarker and an important therapeutic target. The development of novel small-molecule inhibitors of MEX3A or the targeted inhibition of MEX3A expression using methods such as MEX3A blocking peptide, ASO, or AAV would suppress the growth and metastasis of ovarian cancer cells and improve the clinical prognosis of patients. Further studies will be needed to evaluate the clinical value of the MEX3A target for ovarian cancer treatment by constructing PDX mouse models and organoid models of ovarian cancer patients.

In conclusion, we first confirmed the expression, biological roles and regulatory mechanism of MEX3A in ovarian cancer. Through our research, we found that MEX3A was overexpressed in ovarian cancer and associated with poor prognosis in ovarian cancer patients. MEX3A facilitated the proliferation and invasion of ovarian cancer cells by regulating alternative splicing of TIMELESS through the NMD pathway. Taken together, the MEX3A/TIMELESS axis promoted the malignant biological behavior of ovarian cancer cells.

## Materials and methods

### Patients and tumor samples

In our present study, we collected ovarian cancer and fallopian tube (FT) samples from patients who underwent gynecological surgical excision at Qilu Hospital of Shandong University from April 2019 to August 2021. Ovarian cancer specimens were taken from primary ovarian cancer patients who had not previously undergone chemotherapy treatment or surgery. FT tissue was extracted from patients with benign diseases who underwent full hysterectomy and bilateral salpingectomy. We used 34 fresh-frozen ovarian cancer and 18 FT tissues for quantitative real-time PCR (qRT-PCR) and western blotting. The pathological type of ovarian cancer tissues used for qPCR, western blot and immunohistochemistry (IHC) staining experiments in this study was high-grade serous ovarian cancer (HGSOC). Ethics recognition was accepted by the Ethics Committee of Shandong University School of Medicine (SDULCLL2019-1-09).

#### Bioinformatics analysis

The differentially expressed genes data between ovarian cancer and normal ovary tissues of TCGA was from GEPIA ( | Log2FC | ≥ 1, *q* < 0.01) (http://gepia.cancer-pku.cn/). The effect of TIMELESS-205 on the overall survival of ovarian cancer patients was also from GEPIA. A high-priority list of 145 RBPs associated with ovarian cancer aggressiveness was from Linjie Zhao’s study [20]. The differentially expressed genes data between ovarian cancer and normal tissues of TCGA-GTEX was from UCSC Xena (http://xena.ucsc.edu/). The effect of RBPs on clinical prognosis was evaluated by Kaplan–Meier Plotter [37] (http://kmplot.com/). The relative expression of MEX3A in different molecular subtype was from cBioPortal [38] (http://www.cbioportal.org/). The correlation analysis between MEX3A amplification and mRNA expression in ovarian cancer tissues and cell lines was from cBioPortal and Cancer Cell Line Encyclopedia (https://sites.broadinstitute.org/ccle/datasets). Heatmap of differential expressed genes after MEX3A knockdown in ovarian cancer cells was produced by TBtools [39]. David 6.8 (https://david.ncifcrf.gov/) was used to perform the Gene Ontology analysis of differential expressed genes. Venny 2.1.0 online tool was used to perform overlapping analysis (https://bioinfogp.cnb.csic.es/tools/venny/). Sashimi plot was used to visualize the RNA-seq reads mapped to TIMELESS after MEX3A knockdown. TIMELESS transcripts were from Ensemble website (https://asia.ensembl.org/index.html). The protein expression data between ovarian cancer and normal ovary tissues was from Clinical Proteomic Tumor Analysis Consortium (CPTAC).

### Cell culture

The A2780 and HEY cell lines were kind gifts from the laboratory of Dr. Wei. The SKOV3 and HEK293T cell lines were purchased from the Chinese Academy of Sciences (Shanghai, China). A2780 cells were cultured in RPMI 1640 (Gibco, Thermo Fisher Scientific, Inc.) supplied with 10% fetal bovine serum (FBS Gibco, Thermo Fisher Scientific, Inc.). SKOV3 cells were cultured in MacCoy’s5A plus 10% FBS. HEK293T and HEY cells were maintained in DMEM (Gibco, Thermo Fisher Scientific, Inc.) with 10% FBS. The cell lines were cultured in a humidified incubator at 37 °C with 5% CO2. All the cell lines were identified by short tandem repeat (STR) analyzes and mycoplasma testing.

### Lentiviral infection and RNA interference

The MEX3A overexpression lentiviral plasmid was purchased from OriGene (RC215359L3, USA). The shRNA lentiviral plasmids of MEX3A were obtained from Sigma-Aldrich. Lentiviral vectors were packaged in HEK293T cells using psPAX2 and pMD2.G to produce lentiviral particles. Ovarian cancer cells (A2780, SKOV3 and HEY) were infected with lentivirus for 24 h. The stably transfected cell lines were screened out with culture medium containing 2 μg/mL puromycin (Merck Millipore, USA) for 7 days.

Small interfering RNA (siRNA) sequences targeting MEX3A, UPF1 and TIMELESS were purchased from GenePharma (Shanghai, China). Cells were maintained to 30–50% confluence and then transfected with siRNA or nontargeting control (siNC) using Lipofectamine 2000 (Invitrogen, USA). The sequences of siRNAs ere shown in Table S1.

### RNA isolation, qRT-PCR and RT-PCR

TRIzol reagent (Invitrogen, USA) was used to extract the total RNA of the fresh-frozen tissues and cell lines. The RNA was reverse transcribed into cDNA using the PrimeScript RT kit (Takara, JAPAN). qRT-PCR was used to amplify cDNA with SYBR-Green qPCR premix (Takara, Bio, Inc. Japan) on QuantStudio 3 (Applied Biosystems, USA). GAPDH was used as the internal control. The 2 ^−ΔΔCT^ method was used to analyze the qRT-PCR data. RT-PCR (reverse transcription-polymerase chain reaction) was applied to amplify the spliced and unspliced transcripts of TIMELESS mRNA. The primer sequences were listed in Table S2.

### Protein extraction and western blotting analysis

The tissues and cell lines were lysed in RIPA buffer (Beyotime, China). A BCA protein assay kit (Merck Millipore, USA) was used to quantify the protein concentration. The protein samples were separated with SDS–PAGE. Then, the proteins were transferred to PVDF membranes (Merck Millipore, USA) and blocked at room temperature for 2 h with 5% skimmed milk. PVDF membranes containing protein bands were incubated with primary antibodies overnight at 4 °C. The bands were incubated with secondary antibodies for 2 h on the second day. Then, bands were detected with an ECL system (GE Health care). β-actin was used as an endogenous control. Relative protein levels were analyzed using ImageJ 1.47. The primary antibodies used in this study for western blotting assays and immunohistochemistry (IHC) staining were MEX3A (AB_2812582, Invitrogen, USA) and TIMELESS (14421-1-AP, Proteintech, China). An antibody against β-actin (A5441) was purchased from Sigma-Aldrich (USA).

### Cell proliferation assays

Cell proliferation was detected using the MTT (Sigma-Aldrich, USA) method. A total of 800–1000 cells per well were plated in 96-well plates and cultured for 6 days. Four hours after inoculation, 20 μl (5 mg/ml) MTT reagent was added to each well at the same time every day and then incubated in an incubator at 37 °C for another 4 h. Then, the supernatant was removed, and 100 μl DMSO (Sigma-Aldrich, USA) was added to each well. The absorbance at 490 nm was detected by a Varioskan Flash enzyme labeling instrument (Thermo Fisher Scientific, Inc. USA).

### EdU assay

The transfected cells were seeded into 96-well plates (1 × 10^4^ cells) and cultured overnight in a 37 °C incubator. According to the instructions of the BeyoClick™ EdU Cell Proliferation Kit with Alexa Fluor 488, EdU detection was performed. Then, EdU-positive cells were stained with Azide 488 and Hoechst 33342. Images from three random fields of vision were taken under a microscope. The percentage of EdU-positive cells was calculated by the following formula: EdU-positive rate = EdU-positive cell count/(EdU-positive cell count + EdU negative cell count) × 100%.

### Clonogenic assays

To assess the colony formation ability of cells, 800–1000 cells per well were plated in 6-well plates and cultured in incubators at 37 °C for 10–14 days. Then, methanol was used to fix the colonies for 15–30 min and stained with 0.1% crystal violet for 15 min. Finally, we counted colonies (>50 cells) to evaluate the colony formation ability.

#### Nude mice xenograft assay

The animal experiments were approved by the Animal Care and use Committee of Shandong University and complied with its guidelines and policies. Female non-thymic nude mice (BALB/c; 4–6 weeks old) were purchased from Beijing Weitong Lihua and maintained in the specific‑pathogen‑free (SPF) condition. We constructed the sh-Ctrl and sh-MEX3A cell lines in A2780 cells. The cells were subcutaneously injected into each nude mouse. After 3 weeks, the mice were euthanized and dissected, and the tumor was photographed, weighed and calculated. Immunohistochemistry assay was used to observe the expressions of Ki-67 in tumor tissues.

### Invasion and migration assays

To assess the invasion and migration ability of cells, the Transwell chamber (BD Biosciences, USA) was placed in a 24-well plate. In the case of the invasion test, Matrigel (BD Biosciences, USA) was laid in the upper chamber. No Matrigel was coated in the upper chamber for the migration assay. Two hundred microlitres of serum-free medium containing 1 × 10^5^ cells was seeded into the upper chamber of the Transwell, and 700 μl of medium containing 20% FBS was added to the lower chamber. The Transwell chamber was placed in a 37 °C incubator for an appropriate time (6–24 h). The cells in the upper chamber were removed with cotton swabs, and the cells on the lower surface of the chamber were fixed with methanol for 15 min. Crystal violet (0.1%) was stained at room temperature for 15 min. We observed the cell quantities under a light microscope.

### RNA sequencing analysis

We knocked down MEX3 A using specific siRNA in A2780 cells. Total RNA was isolated with TRIzol reagent according to the manufacturer’s protocol. Next-generation sequencing technology was performed by Annoroad Genomics Co., Ltd, China. The raw data was uploaded to GEO database (GSE189415). Genes with fold change >1.2 and fold change <0.8 (*P*_adj_ value < 0.05) were considered differentially expressed genes (DEGs).

### RNA immunoprecipitation assay (RIP)

RIP assays of MEX3A using monoclonal anti-Flag antibody (F1804) from Sigma-Aldrich (with IgG as control) were performed using a Magna RIP RNA-Binding Protein Immunoprecipitation Kit (Millipore, 17–700) according to the manufacturer’s instructions to analyze the interaction between MEX3A protein and TIMELESS mRNA. qRT-PCR analysis was used to detect the level of TIMELESS mRNA in the immunoprecipitated complex.

### Statistical analysis

Statistical analysis was performed using GraphPad Prism 5 (GraphPad Software, CA, USA). Student’s *t*-test was chosen to analyze the difference in quantitative data. Results represent the mean ± SD of three independent experiments. Experimental data *P* < 0.05 (*P < 0.05, **P < 0.01) were considered statistically significant.

## Supplementary information

Supplementary Figures and tables

Original images of WB and RT-PCR

author contribution

checklist

## Data Availability

The datasets used and/or analyzed during the current study are available from the corresponding author on reasonable request.
