# Supplementary material for: MEX3A promotes the malignant progression of ovarian cancer by regulating intron retention in TIMELESS
Source: Cell Death Dis. 2022 Jun 17;13(6):553. doi: 10.1038/s41419-022-05000-7 (PMC9205863; doi:10.1038/s41419-022-05000-7)
Supplement: Supplementary file 1 — Supplementary Figures and tables [file 41419_2022_5000_MOESM1_ESM.docx]

**Supplementary Figures**


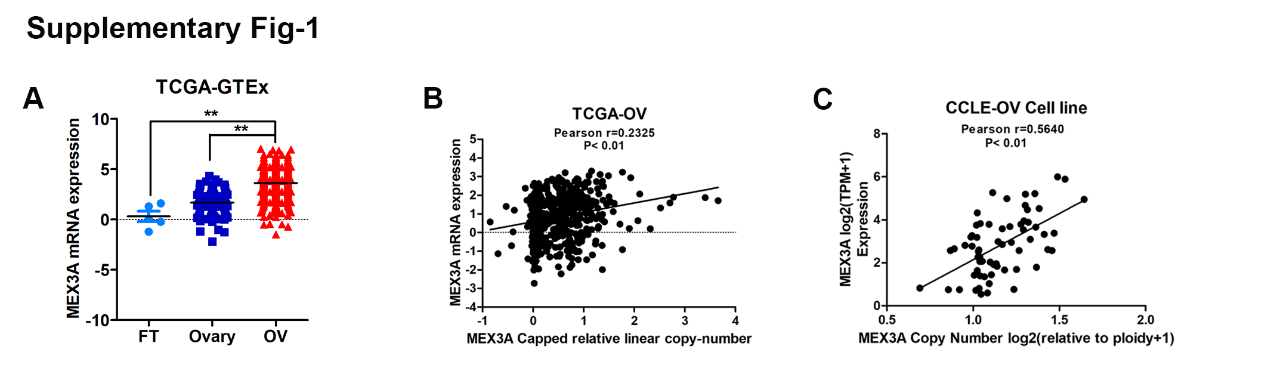


(A) Relative mRNA expression of MEX3A in ovarian cancer (n=426), normal ovary (n=88) and fallopian tube (n=5) tissues from TCGA-GTEx. (B, C) Correlation analysis between MEX3A amplification and mRNA expression in TCGA ovarian cancer samples (n=528) and CCLE ovarian cancer cell lines (n=64 pairs). P value was obtained by Unpaired t-test (C, D, E and H). *P < 0.05, **P < 0.01.

**Supplementary tables**

Table S1. si-RNA sequences used in this study.

Table S2. Primer sequences used in this study.

Table S3. Correlation analysis between MEX3A expression and clinical parameters.

**Table S1. si-RNA sequences used in this study.**

| Method | Name | Sequence (5’-3’) |
| --- | --- | --- |
| si-RNA | si-MEX3A#1 | GCAAGGCTGCAAGATTAAGTT |
| si-RNA | si-MEX3A#2 | GCAAGAUCCUCGAGUACAATT |
| si-RNA | si-TIMELESS#1 | AGAAGAGAAGGAAGAAGAATT |
| si-RNA | si-TIMELESS#2 | GCCUACAUGUGCUAGAGAUTT |
| si-RNA | negative control | UUCUCCGAACGUGUCACGUTT |
|  |  |  |

**Table S2. Primer sequence used in this study.**

| Method | Name | Sequence (5’-3’) |
| --- | --- | --- |
| qPCR | GAPDH-F | GGTCTCCTCTGACTTCAACA |
| qPCR | GAPDH-R | GTGAGGGTCTCTCTCTTCCT |
| qPCR | MEX3A-F | CAGCAGCAACACCACGGAGTG |
| qPCR | MEX3A-R | CGGTGTCTTGATGTAGGTGTTGG |
| qPCR | TIMELESS-F | TCTGATCCGCTATTTGAGGCA |
| qPCR | TIMELESS-R | GGCAGAAGGTCGCTCTGTAG |
|  |  |  |

**Table S3. Correlation analysis between MEX3A expression and clinical parameters.**

| Characteristics | Number of cases | MEX3A expression | | P value |
| --- | --- | --- | --- | --- |
|  |  | Low | High |  |
| Age |  |  |  | 0.5245 |
| <50 | 33 | 11 | 22 |  |
| ≥50 | 78 | 31 | 47 |  |
| CA125(U/ml) |  |  |  | 0.5766 |
| <1000 | 65 | 26 | 39 |  |
| ≥1000 | 46 | 16 | 30 |  |
| FIGO stage |  |  |  | 0.5984 |
| I+II | 21 | 9 | 12 |  |
| III+IV | 90 | 33 | 57 |  |
| Ascites(ml) |  |  |  | 0.0388 |
| <1000 | 47 | 23 | 24 |  |
| ≥1000 | 64 | 19 | 45 |  |
