# Supplementary figures and images for: MEX3A promotes the malignant progression of ovarian cancer by regulating intron retention in TIMELESS
Source: Cell Death Dis. 2022 Jun 17;13(6):553. doi: 10.1038/s41419-022-05000-7 (PMC9205863; doi:10.1038/s41419-022-05000-7)

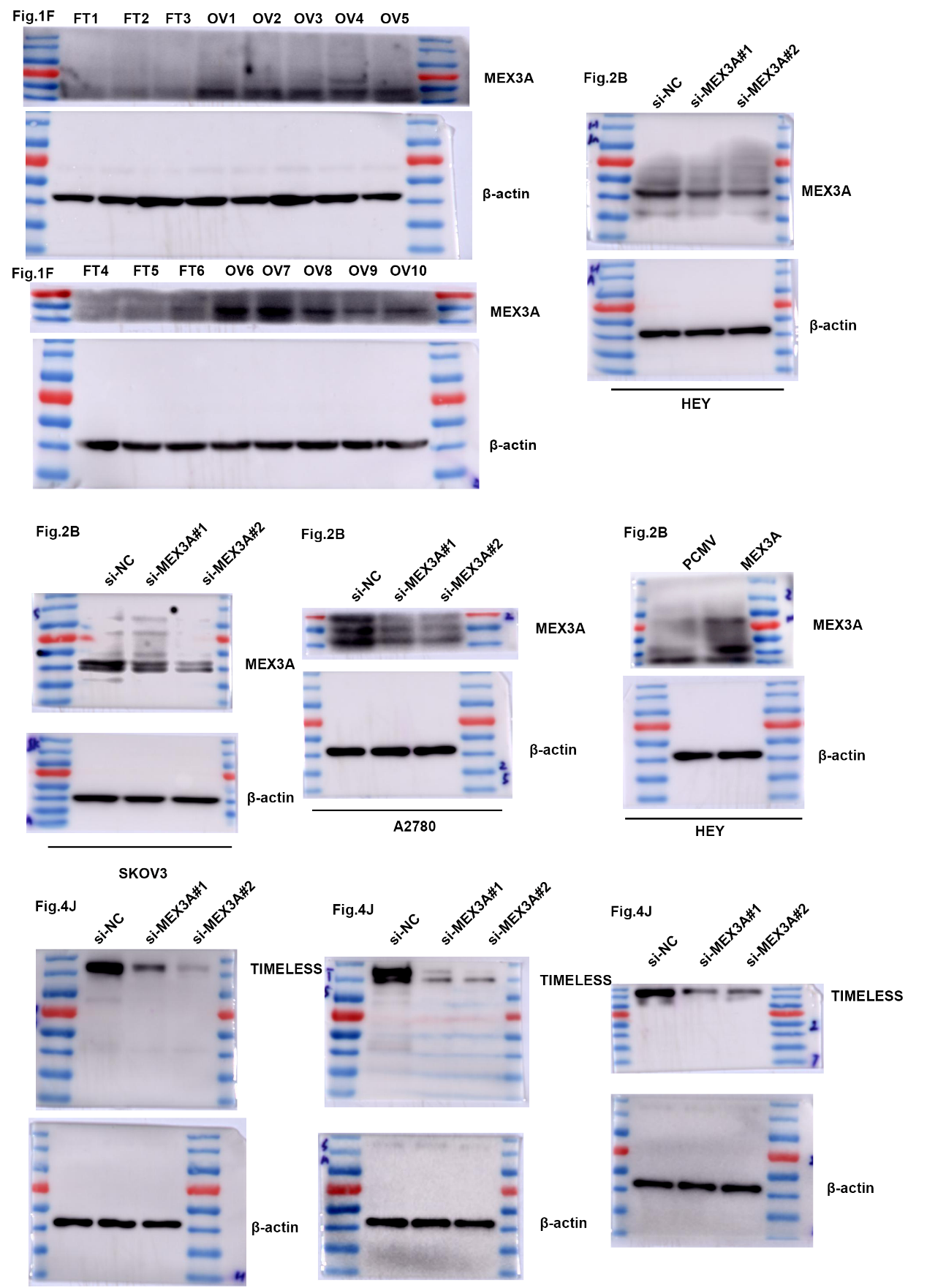


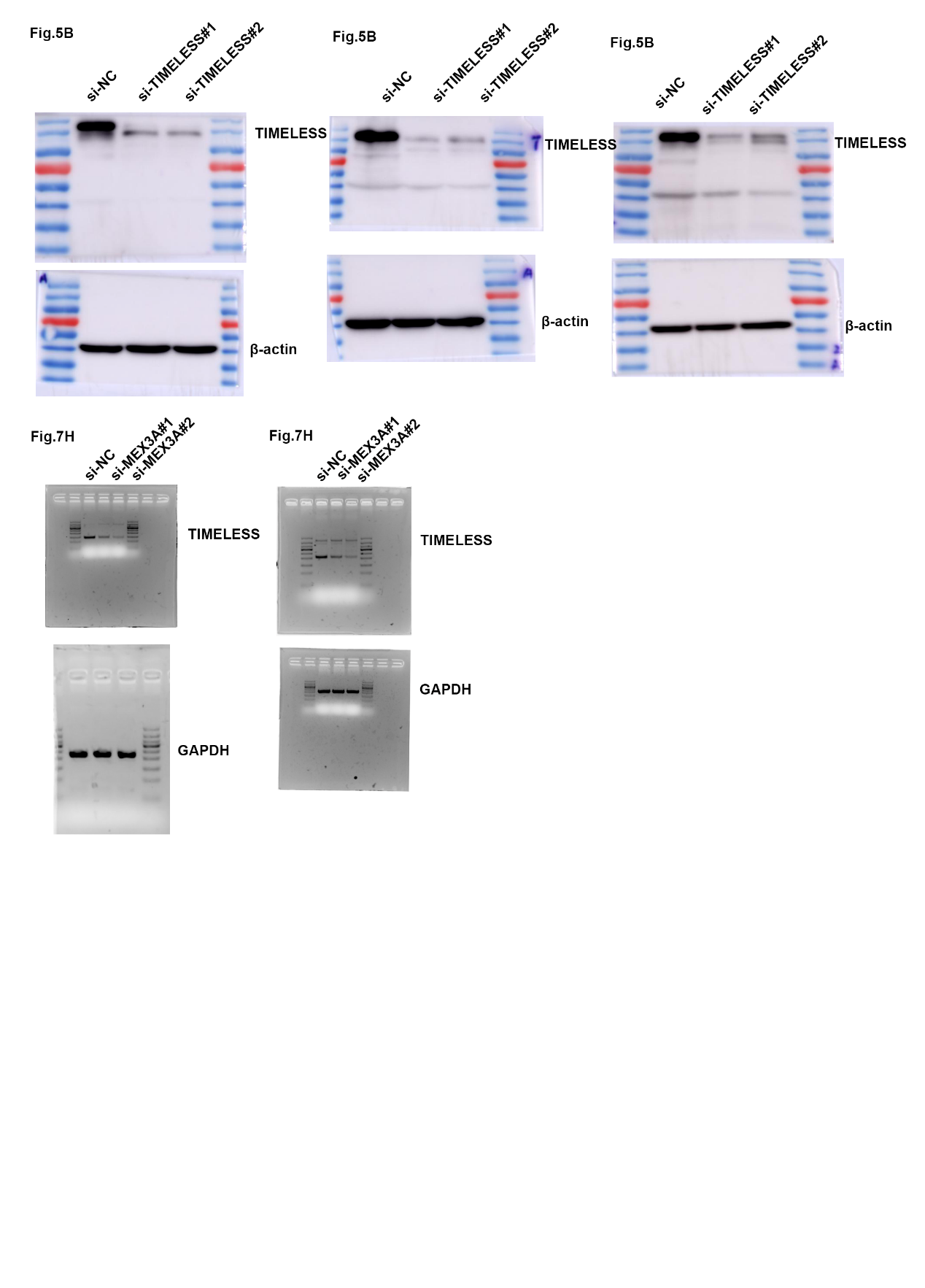

Supplement: Supplementary file 2 — Original images of WB and RT-PCR [file 41419_2022_5000_MOESM2_ESM.docx]
