# Supplementary material for: MEX3A promotes the malignant progression of ovarian cancer by regulating intron retention in TIMELESS
Source: Cell Death Dis. 2022 Jun 17;13(6):553. doi: 10.1038/s41419-022-05000-7 (PMC9205863; doi:10.1038/s41419-022-05000-7)
Supplement: Supplementary file 3 — author contribution [file 41419_2022_5000_MOESM3_ESM.pdf]

DECLARATION OF CONTRIBUTIONS TO ARTICLE

ADMC

Manuscript Number: CDDIS-21-5134

Journal Name: *Cell Death & Disease* (the 'Journal')

Proposed Title of the Contribution:

MEX3A promotes the malignant progression of ovarian cancer by regulating intron retention in TIMELESS (the 'Contribution)

Author(s):

Fangfang Li, Chen Zhao , Yuchao Diao, Zixiang Wang, Jiali Peng, Ning Yang, Chunping Qiu, Beihua Kong,Yingwei Li (the 'Authors')

For all *CDDis* articles, each person named as an author in the published version must be able to show he or she has contributed substantially to the article.

Authorship credit should be based on 1) substantial contributions to conception and design, acquisition of data, or analysis and interpretation of data; 2) drafting the article or revising it critically for important intellectual content; and 3) final approval of the version to be published. Authors should meet conditions 1, 2 and 3.

Any person who cannot be shown to have made a substantial contribution to the article cannot be listed as an author in the final version. The name of any person who is deemed to have made a minor contribution can, however, appear in the Acknowledgments section of the article.

Please complete the table below to indicate the contributions of all named authors to the manuscript.

Author Full Name:	Specification of Contribution to the Manuscript:
Fangfang Li	Acquisition, analysis and interpretation of data; Drafting and final approval of the article
Chen Zhao	Analysis and interpretation of data; Technical support; Revision and final approval of the manuscript
Yuchao Diao	Interpretation of data; Technical support; Revision and final approval of the manuscript
Zixiang Wang	Analysis and interpretation of data: Revision and final approval of the manuscript
Jiali Peng	Analysis and interpretation of data: Revision and final approval of the manuscript
Ning Yang	Technical support; Revision and final approval of the manuscript
Chunping Qiu	Nude mice xenograft assay
Beihua Kong	Conception and design; Revision of the manuscript; Administrative, and material support; Final approval and Study supervision
Yingwei Li	Conception and design; Revision of the manuscript; Administrative, and material support; Final approval and Study supervision

Please complete the table below to indicate the contributions of all named authors to the figures.

Figure 1:

F.L generated the data and prepared panel E and F; Y.L generated the data and prepared panel A, B, C and D; Z.W generated other data; J.C labelled the image; N.Y provided the clincal samples.

Figure 2:

F.L generated the data; J.P labelled the image. Y.L assembled the figure.

Figure 3:

F.L and C.Z generated the data and assembled the figure.

Figure 4:

Z.W generated the data; Y.L assembled the figure.

Figure 5:

F.L generated the data and Y.L assembled the figure.

Figure 6:

F.L generated the data and Y.L assembled the figure.

Signed for and on behalf of the Author(s):	Print Name:	Date:
Fangfang Li Chen Zhao Yuchao Diao, Zixiang Wang Jiali Peng	Fangfang Li, Chen Zhao , Yuchao Diao, Zixiang Wang, Jiali Peng, Ning Yang, Yingwei Li, Beihua Kong	2022/02/18

Ning Yang Yingwei Li Beihua Kong

Chunping Qiu
